# Bioenhancer Assessment of Black Pepper with Turmeric on Self-Reported Pain Ratings in Adults: A Randomized, Cross-Over, Clinical Trial

**DOI:** 10.3390/nu18020223

**Published:** 2026-01-10

**Authors:** Leandra Durham, Robert A. Oster, Matthew Ithurburn, Chelsi Reynolds, James O. Hill, Daniel L. Smith

**Affiliations:** 1 Department of Nutrition Sciences, University of Alabama at Birmingham, Birmingham, AL 35233, USA; 2 Department of Medicine, University of Alabama at Birmingham, Birmingham, AL 35294, USA; 3 Center for Clinical and Translational Science, University of Alabama at Birmingham, Birmingham, AL 35233, USA; 4 American Sports Medicine Institute, Birmingham, AL 35205, USA; 5 School of Health Professions, University of Alabama at Birmingham, Birmingham, AL 35294, USA; 6 Nutrition Obesity Research Center, University of Alabama at Birmingham, Birmingham, AL 35233, USA; 7 Integrative Center for Healthy Aging, University of Alabama at Birmingham, Birmingham, AL 35294, USA; 8 Nathan Shock Center of Excellence in the Basic Biology of Aging, University of Alabama at Birmingham, Birmingham, AL 35294, USA

**Keywords:** pain, chronic pain, spices, culinary spices, experience sampling methodology, ecological momentary assessment, nutrition intervention

## Abstract

Background: Chronic pain, which affects over 50 million adults in the United States, has stimulated growing interest in natural and nutrition-based remedies as adjuncts to pharmacologic therapies. Evidence suggests that turmeric and related extracts (i.e., curcuminoids) may provide pain relief, albeit often at levels above dietary ranges, while piperine from black pepper exhibits bioenhancer characteristics of relevance with dietary exposures. Objective: To test the effectiveness of dietarily relevant amounts of turmeric with and without black pepper on self-reported pain ratings among adults with chronic pain. Methods: A randomized, crossover clinical trial tested the effectiveness of turmeric only (one of three amounts within culinary ranges) or turmeric with black pepper to influence pain in adults ≥ 40 years of age. Participants (*n* = 30, with moderate pain: 4–7 on 0–10-point scale) were enrolled in a 21-day trial, and an experience sampling methodology approach was used. Participants were prompted to report current pain using the numeric pain rating scale (NPRS; 0–10) via text message three times per day for the full study period. Data were averaged and analyzed via linear mixed effects models for repeated measurements. Results: Pain ratings from baseline to week 3 were reduced and statistically significant (*p* < 0.001) but not statistically different between groups. The provided turmeric, both with and without black pepper, and varying amounts of turmeric (300 mg, 1 g, and 3 g, *n* = 10 participants/amount) did not show statistically significant differences in pain ratings (*p* = 0.157 and *p* = 0.338, respectively). Conclusions: Consuming dietarily relevant amounts of turmeric, either alone or with black pepper, appears to improve average pain ratings. This result suggests a feasible dietary option for further study of nutritional interventions for chronic pain management.

## 1. Introduction

Chronic pain is considered pain that lasts three months or longer, and it is estimated to affect 51 million American adults [1,2,3]. Pain is one of the most common secondary conditions associated with non-communicable chronic diseases and disabilities. This can be burdensome, reducing the quality of life and ability to participate in activities of daily living (ADL) for individuals who experience moderate to severe chronic pain [1,3]. Managing chronic pain can involve a variety of treatment options and focuses on patients’ perceptions of success in reducing reported pain severity and frequency.

Pharmaceutical options for pain management include opioids, non-steroidal anti-inflammatory drugs, corticosteroids, and analgesics [1,2,3]. Pharmaceutical options can be associated with significant side effects, risk of addiction, financial burden, and potential contraindications, highlighting some of the reasons why patients have reported interest in non-pharmacological approaches or adjunct therapies for chronic pain management [4]. Approaches that have been tested and recommended clinically include cold or heat therapies, behavioral therapies, acupuncture, exercise interventions, and nutrition interventions [4].

While exercise and nutrition interventions are recommended for pain management, chronic pain has been reported as a barrier to engaging in healthy lifestyle behaviors such as physical activity and healthful eating [4,5,6]. This can present an inherent challenge for adults suffering from chronic moderate or severe pain. Opportunities exist to study the potential benefits of herbs and spices in their utility for pain management as alternatives or adjuvants to over-the-counter (OTC) medications. Finding low-barrier ways, such as a nutrition intervention that utilizes the addition of herbs or spices to participants’ normal eating patterns, to help reduce pain through nutrition may help individuals increase their daily physical activity and thus further reduce pain levels.

Anti-inflammatory diet patterns have been recommended for use in chronic pain management [7]. Anti-inflammatory diets typically focus on the inclusion of plant-based and minimally processed foods as well as limiting refined carbohydrates, ultra-processed meats, and sometimes dairy foods. Dietary modifications such as these can require significant behavior changes, resulting in obstacles to adoption and implementation [8]. Specific dietary ingredients or factors, such as herbs and spices, often possess bioactive components with anti-inflammatory or pain-relieving effects and offer less extreme dietary approaches to potentially help reduce chronic pain. Spices that have been used and tested for pain relief include cinnamon, ginger, turmeric, and black pepper [9,10,11,12]. Many of these agents have been investigated in people with osteoarthritis pain and at relatively high intake amounts, with less of a focus on general moderate chronic pain and dietary feasible levels [11,13].

Turmeric has been widely studied for pain reduction, focusing on the bioactive component of curcumin/curcuminoids [14,15]. The pain-relieving effects of turmeric with bioactive curcuminoids are attributed to the anti-inflammatory and antioxidant properties. This includes inhibition of pro-inflammatory molecular mediators (e.g., COX-2, cytokines) and modulation of signaling pathways [16,17,18]. Due to curcuminoids’ low bioavailability, meaning the amount and rate at which the body absorbs the compound, highly concentrated extracts have been utilized to increase the bioactive compounds above dietary levels, with demonstrated benefits for pain relief [11,13]. An alternative approach to increase bioavailability is the co-consumption of bioenhancers [11,19]. Bioenhancers are generally considered substances that are capable of increasing either the “efficacy” or the “availability” of biologically active compounds in a system [19,20,21,22,23,24,25]. Several spices have been identified for their bio-enhancing properties, including black pepper. Black pepper, which contains the bio-enhancing compound piperine, estimated to be 2–7% by dry weight of the ground spice, is one of the most common spices in culinary use and has been demonstrated to increase the bioavailability of curcuminoids by up to 154% [20,21,22,24,26,27].

The goal of our present study was to address a gap in studies assessing the potential analgesic benefits of dietarily relevant amounts of turmeric, considering interactive effects with the often co-consumed spices, specifically black pepper, and utilizing real-time, longitudinal assessment of self-reported pain. The aim of this study was to investigate the effect of turmeric in one of three amounts, all within dietary ranges, both with and without black pepper, on improving pain among adults suffering from chronic pain. We hypothesized that the combination of black pepper with turmeric would significantly decrease pain ratings as compared to turmeric alone. Additionally, the study aimed to assess whether pain reduction through the intake of turmeric alone or turmeric with black pepper alters daily physical activity.

## 2. Materials and Methods

### 2.1. Statement of Ethics and Approval

This study was conducted in accordance with the Declaration of Helsinki and approved by the University of Alabama at Birmingham (UAB) Internal Review Board (IRB) and conformed to the standards of use of human subjects in research as set by the IRB (IRB protocol#-300005070 approved 9 December 2021). All participants were informed of the study purpose, procedures, and potential risks before written consent was obtained. The trial was registered on clinicaltrials.gov (NCT05206266) and was conducted at UAB.

### 2.2. Participants

Participants were recruited through the UAB School of Health Professions (SHP) Research Collaborative, the UAB Health System (via radio advertisements, public service announcements, notices in newspapers, campus announcements and emails, flyers, and social media postings), and a partnership between the UAB Department of Nutrition Sciences and the UAB Department of Family and Community Medicine clinic, which holds a registry of individuals interested in being contacted for research participation. Participants were eligible if they were aged 40 years or older. Participant inclusion criteria included self-reported moderate chronic pain of 4–7 on a numeric pain rating scale ranging from 0 to 10, with 0 representing no pain and 10 representing the worst possible pain, and having no recent acute changes in pain. The inclusion and exclusion criteria for participating in this study are reported in Table 1.

### 2.3. Experimental Design and Study Overview

**Figure 1:**
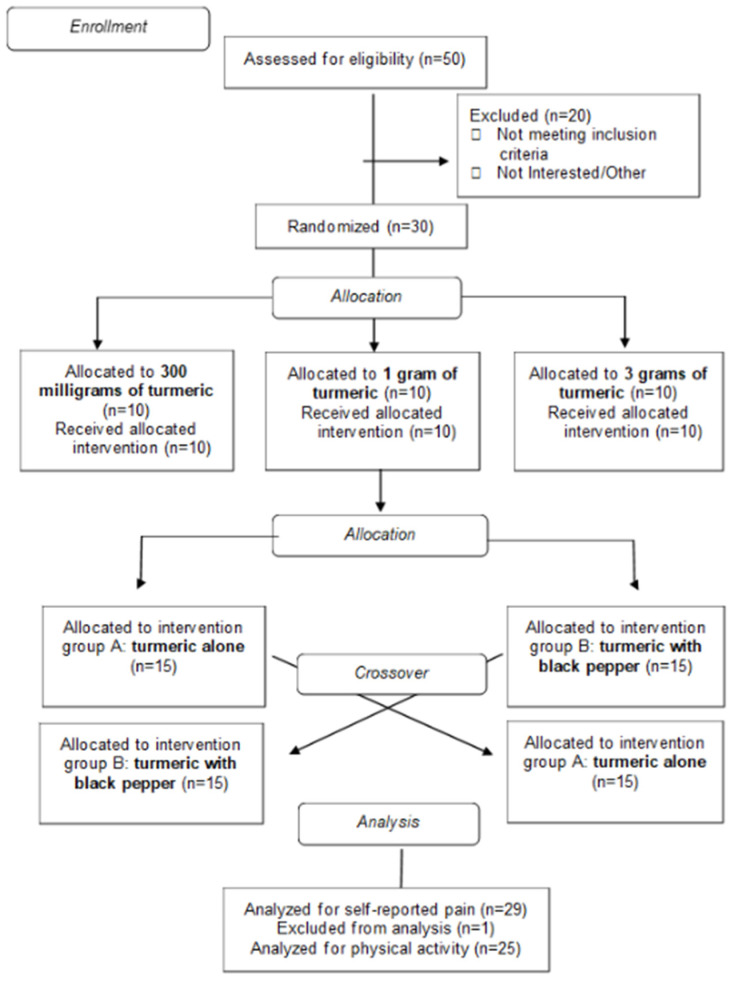
CONSORT Flow Diagram. Includes allocations of treatment, spice amount, and participant analyzed. Participants were excluded from analysis due to discontinuing participation in the study (*n* = 1) and missingness of accelerometry data (*n* = 4).

A randomized 21-day double-blind crossover clinical trial was conducted to test the effectiveness of turmeric versus turmeric with black pepper on self-reported pain ratings (*n* = 30 individuals with chronic pain). Randomization of treatment was performed using a computer number sequence generator, and allocations were blinded to the study team and participants. The study team was unblinded after the analysis was performed and the results were ready for interpretation. Participants were enrolled, their baseline pain rating was recorded, and then they were randomized into one of two sequences: receiving turmeric alone for the first seven days of the study or receiving turmeric with black pepper for the first seven days of the study. All participants were assigned a seven-day washout and then crossed over to receive the alternate treatment of turmeric with black pepper or turmeric alone for the last seven days of the study (see Figure 1).

The spices were purchased commercially (Ground Turmeric, Organic (non-GMO), McCormick^®^; Pure Ground Black pepper (non-GMO), McCormick^®^; purchased from Amazon.com, 17 February 2022) and stored in temperature and light-controlled conditions (i.e., room temperature and lighting, avoiding direct sunlight). The sealed containers were provided for encapsulation by a local pharmacist for blinding purposes. Encapsulated spices were provided to participants in either blister packs or sealed bags labeled with daily consumption instructions. Turmeric was provided as one of three different amounts (300 mg, 1 g, or 3 g; *n* = 10 participants/turmeric amount) and black pepper as one amount when present (300 mg). Participants were instructed to consume the encapsulated spices with a lunch meal and to avoid additional consumption of black pepper and turmeric for the study duration.

Participants engaged in longitudinal data collection by reporting their pain ratings three times per day through a text message software. Participants were also asked to wear accelerometers during both sleep and wake hours for the 21-day study duration. At baseline, participants were asked to fill out a brief survey to further understand their pain in relation to intensity and anatomical location.

### 2.4. Nutrition Intervention

Both the turmeric and black pepper were sourced through retail venues and were given to participants as capsules at the beginning of the study, labeled for each week. Participants were assisted in selecting seven lunchtime meals from a local meal delivery service (Fit Five Meals), which were provided in triplicate sets for the three weeks of the study (*n* = 21 meals in total, three sets of seven meals). Participants were instructed to consume the same meal consistent with each day of the week (e.g., meal 1 consumed on study day 1 also needed to be the meal consumed on study days 8 and 15, respectively). Participants were instructed to consume the encapsulated spices with the provided meals. Meals were labeled accordingly, with encapsulated spices having daily consumption designations for the 21-day study. Participants were encouraged to maintain their normal dietary practices, with no other dietary/nutrition modifications requested.

#### Self-Reported Pain Ratings

Baseline pain ratings were recorded as part of the screening protocol. For the duration of the study, an experience sampling methodology (ESM) was used to obtain self-reported numeric pain ratings three times per day through text messages and captured via SurveySignal software (3/day for 21 days) [28,29,30]. The text messages were sent between 8:00 a.m. and 8:00 p.m., around breakfast, lunch, and dinnertime, but exact times varied throughout the day. The pain ratings were averaged for the course of the week for each participant and study period, providing the mean pain rating for each week to be used in the statistical analyses. The prompt for the self-reported numeric pain rating was consistent throughout the study period: “On a scale of 0 to 10, with 0 being no pain at all and 10 being the worst pain imaginable, how would you rate your pain RIGHT NOW?” A reminder text was sent after 15 min if no response was captured following the initial text message. An additional pain inventory survey was used to capture the location of pain via Qualtrics.

### 2.5. Physical Activity Monitoring

Participants were provided a wGT3X-BT ActiGraph accelerometer (ActiGraph, Pensacola, FL, USA) with instructions to wear it on their non-dominant wrist or waist during both sleeping and waking hours for the duration of the study [31,32]. Data were downloaded from the accelerometers and refined, excluding spurious data, and then minute-by-minute activity counts were calculated. A total of *n* = 25 participants were included in the analysis of physical activity measures due to missing accelerometry data from *n* = 4 participants. Missing data were due to both a lack of device wear and failure to return the accelerometer. Physical activity was defined based on intensity categories as the total recorded length of time in the light, moderate, vigorous, and very vigorous categories. The Freedson method was used to process the accelerometer data and determine bouts of physical activity within each intensity level [33]. Physical activity time was averaged for each week of the study period and was calculated for each participant to be used in the statistical model. Valid minimum wear time was defined as >480 min per day for at least four days of the study duration, and participants who had valid wear time for at least one week of the study period were included in the analysis. Of the 25 participants included in the analysis, 3 participants had valid wear time for week 1 and an invalid wear time of 0 min for week 3. However, due to the valid wear time for week 1, the three participants were included in the average wear time evaluation. Average wear time for each participant and each study period was calculated for each week of the study period.

### 2.6. Statistical Analyses

Statistical analyses were conducted using SAS software version 9.4 [34]. One participant did not continue with the study after one day of participation due to complaints of gastrointestinal discomfort and was excluded from the analysis. Participant baseline characteristics were summarized using descriptive statistics (mean and SD) and were assessed using a one-way analysis of variance (ANOVA) for continuous variables and chi-square tests for categorical variables.

To test the primary outcome of the effects of turmeric alone or turmeric with black pepper on self-reported numeric pain ratings, linear mixed effects models for repeated measurements were used. Prior to analyzing with the linear mixed effects models, the self-reported pain ratings were averaged for each week of the study period for each participant. Normality and constant variance were assessed using q-q plots to use for a visual check of residuals. To test secondary outcomes of physical activity changes and turmeric amounts, linear mixed effects models for repeated measurements were used. These models included treatment (turmeric alone, turmeric with black pepper), time (baseline, week 1, and week 3), and the treatment by time interaction, with a random intercept for participants.

The primary and secondary outcomes models were estimated using restricted maximum likelihood (REML), and an unstructured covariance matrix was assumed. All linear mixed effects models were run using SAS’s PROC MIXED. Linear mixed effects models are robust against missing data, as they can accommodate unbalanced data patterns [35,36]. Therefore, all observable values in the dataset are included in the analysis; no imputations are performed for missing values. Both pain and physical activity data had missing values due to a lack of response to pain inquiry text messages or a lack of accelerometer wear time.

Power was calculated using G*Power version 3.1.9.7. Using a mixed models statistical test, a power of 80%, and an alpha level of 0.05, a sample size of *n* = 30 participants should detect an effect size of 0.5 between the two groups as being statistically significant. Thus, this study was adequately powered to detect moderate effect sizes as being statistically significant, which has been shown to be a “typical” effect size for pain assessments [37,38,39]. Clinically meaningful results were analyzed as either a 2-point pain reduction on the 0–10 numeric pain rating scale or a 30% reduction from baseline, due to previous studies that show this change as a reflection of “much improved.” Comparatively, a 1-point pain reduction or a 28% reduction has been reported as noticeable but not meaningful [37,39].

## 3. Results

**Figure 2:**
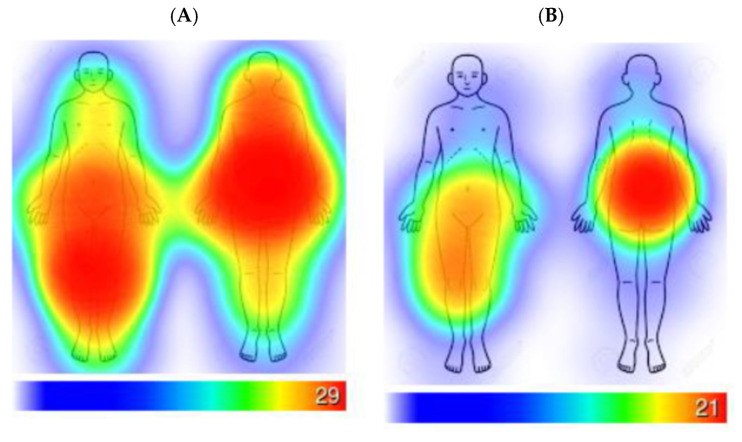
Heatmaps for Reported Pain. Participants could select any region for the site of pain for both front and back anatomical features, which included back and front, legs, knees, hips, hands, arms, spine, shoulders, calves, thighs, head, neck, etc. (**A**) Heatmap illustrating areas participants reported feeling pain when prompted “select the areas you feel pain.” The heatmap includes voluntary responses that represent *n* = 18 out of the total sample of *n* = 29 participants who provided one or more responses about pain locations. (**B**): Heatmap illustrating areas participants reported hurting the most when prompted to “select the area that hurts the most.” Heatmap includes voluntary responses that represent *n* = 18 out of the total sample of *n* = 29 participants who provided one or more responses about pain locations.

As reported in Table 2, study participants were mostly middle-aged adult females, with an average BMI in the obese category. Self-reported numeric pain ratings reflect the recruitment target population. As shown in Figure 2, participants identified the anatomic regions of the lower back, hips, and knees as the sites of the most pain.

### 3.1. Turmeric Alone Versus Turmeric with Black Pepper

**Figure 3:**
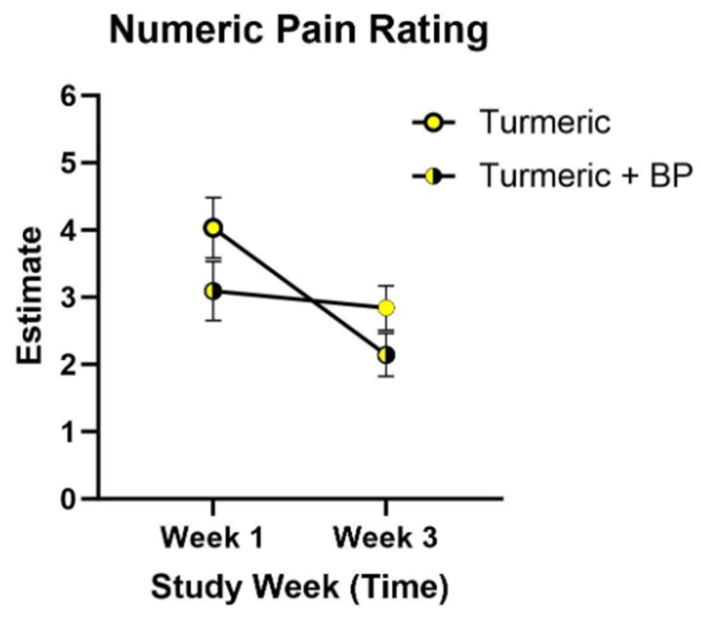
Self-reported pain ratings of the cross-over clinical trial. Self-reported numeric pain ratings by participants who received turmeric alone and those who received turmeric with black pepper in week 1 (*n* = 29) and were subsequently crossed over to receive turmeric alone or turmeric and black pepper in week 3 (*n* = 29). Data were analyzed using linear mixed effects models for repeated measurements (*p* = 0.157). Values are the least square means estimate and standard error. Abbreviations: BP, black pepper.

The comparison of pain ratings over time by treatment is presented in Figure 3. Pain ratings for participants receiving turmeric and turmeric with black pepper changed significantly from baseline to week 3 (*p* < 0.001). Interaction effects of treatment and study periods were analyzed but were not significant (*p* = 0.5508) and were subsequently removed from the statistical model. Self-reported pain ratings were reduced over the course of the study duration for those allocated to both turmeric alone and turmeric with black pepper but were not found to be significantly different between the time periods depending on the presence of black pepper (*p* = 0.157).

### 3.2. Physical Activity

**Figure 4:**
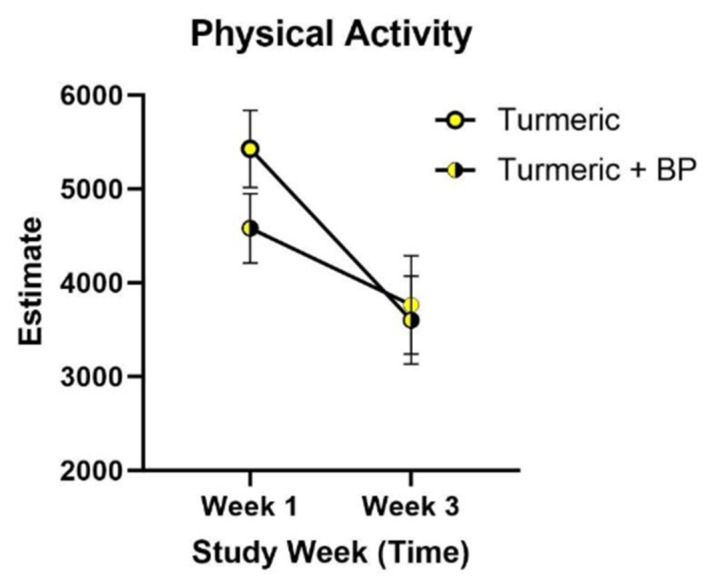
Physical activity minutes per week by treatment group. Physical activity reported by week for participants based on exposure to turmeric alone or turmeric with black pepper in the cross-over design, week 1 (*n* = 25) and week 3 (*n* = 21). Physical activity includes the total minutes of measured light, moderate, vigorous, and very vigorous activity and was analyzed using linear mixed effects models for repeated measurements (*p* = 0.3875). Values are the least square means estimate and standard error. Abbreviations: BP, black pepper.

Average wear time was analyzed for each week of the study period: week 1, 688.7 min per week; week 2, 562.2 min per week; and week 3, 514.8 min per week. Physical activity based on treatment allocation and turmeric amount allocation was not statistically significant (*p* = 0.3875), as shown in Figure 4. Physical activity minutes per week for participants allocated by spice amount received was analyzed and was not statistically significant (*p* = 0.0646).

### 3.3. Turmeric Amount Comparisons

**Figure 5:**
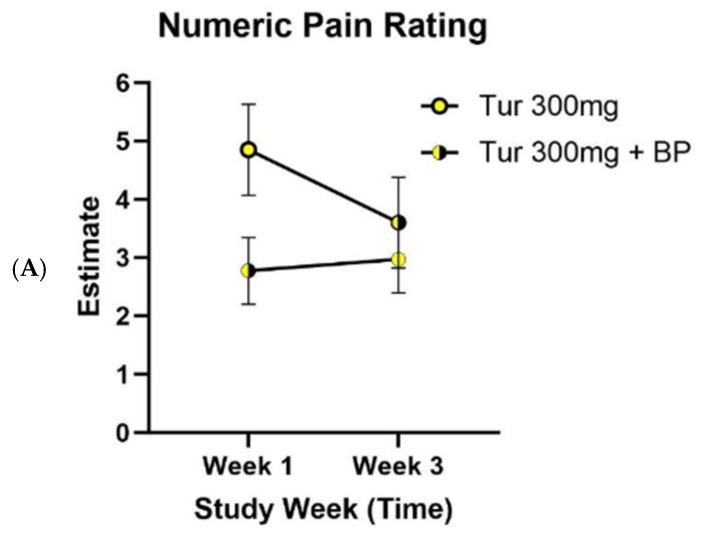

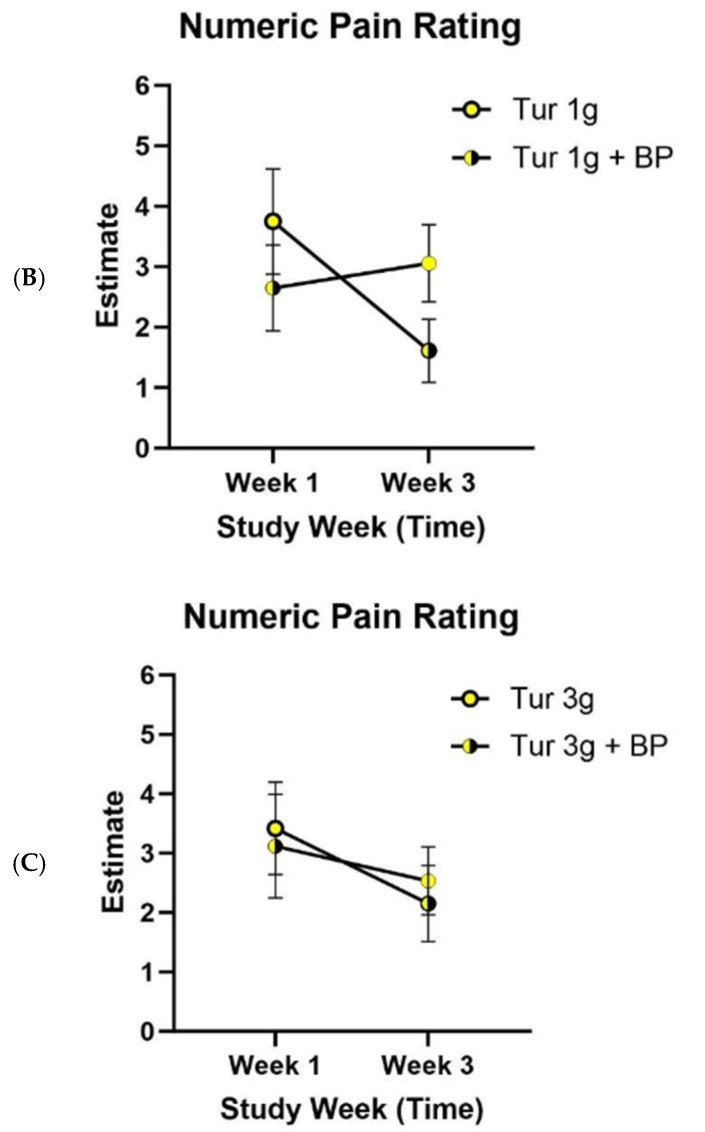
Self-reported pain ratings by week for each allocation of spice amount received by turmeric and turmeric with black pepper intervention within the cross-over design. (**A**) participants receiving 300 mg (*n* = 10) of turmeric with and without black pepper, (**B**) participants receiving 1 g (*n* = 9) of turmeric with and without black pepper, and (**C**) participants receiving 3 g (*n* = 10) of turmeric with and without black pepper. Panels shown were analyzed using linear mixed effects models for repeated measurements. Values are the least square means estimate and standard error. Abbreviations: BP, black pepper; mg, milligram; g, gram.

The comparison of pain ratings over time by turmeric amount and treatment allocation are reported in Figure 5A–C. Pain ratings over time were statistically significant (*p* < 0.001). The interaction effects of the groups of turmeric amounts and study periods were analyzed but were not statistically significant (*p* = 0.5701) and were removed from the statistical model. Despite the absolute numeric values for the self-reported pain values among the turmeric amounts tested at the high-end (3 g) and low-end (300 mg) of consumption (observed particularly at week 1, Figure 5A–C), no statistically significant differences were observed among the three different turmeric amounts (300 mg, 1 g, 3 g; *p* = 0.338).

## 4. Discussion

This study, using commercially available ground spices in culinarily relevant amounts, found that while turmeric with black pepper did reduce self-reported pain levels over time, it was not significantly different from turmeric alone, rejecting the a priori hypothesis. Similarly, higher culinary amounts of turmeric, 1 g and 3 g, showed numerically lower self-reported pain levels over time, but these were not significantly different from the lower culinary amount of 300 mg. All turmeric amounts and turmeric with black pepper showed a reduction in self-reported pain levels over the study period, suggesting that consistent intake of culinary relevant amounts of turmeric and turmeric with black pepper are feasible for improvements in pain reduction.

The previous literature has shown that curcuminoids in various forms of extracts can be as effective in pain reduction as pharmaceutical medications [40]. This literature raises questions as to whether culinarily relevant amounts of turmeric would be effective in reducing pain due to the low bioavailability of its active component(s) [13,41,42]. However, with few studies using culinary spices to test bioavailability, it was unknown if consumption as a ground, dietary spice, particularly in combination with other spices commonly utilized in cultural cuisines, helps the active component show higher bioavailability and accompanying health benefits. These findings suggest that high-potency extracts may not always be necessary for some health benefits previously reported with turmeric [11,40,42,43].

Despite the improvement in self-reported pain, physical activity as measured by wearable accelerometry was not shown to increase with the use of turmeric or turmeric with black pepper over time. The average wear time for the first week of the study was higher for all participants as compared to the third week of the study period. This could have impacted the results found for physical activity. The underlying relationship between chronic pain and physical activity is well established, while the impact of relatively rapid and acute changes in pain as an influence on daily physical activity levels in this demographic is less understood [5,44]. Considering the missingness of the accelerometry data and the study sample size, any extrapolation to broader populations’ durations of measures should be cautiously engaged and carefully considered for future study designs. Future studies could incentivize participants to increase accelerometer wear time by using behavioral change techniques such as reminder texts through similar text message platforms as used in this intervention. Additionally, future studies could consider including an exercise component to better understand the relationship between acute pain relief and physical activity engagement.

### 4.1. Strengths

Strengths of this study include the utilization of self-reported pain using ESM, double blinding of treatment and amount consumed, retention of the study population sample, and consistent meal intake with time-of-day delivery of encapsulated spices. The ESM is a useful way to capture measures that can vary throughout the day or week, like pain, allowing the study to capture nuances of daily chronic pain versus a single-baseline and single-time-point outcome measure [29,30]. The encapsulation of the turmeric or turmeric with black pepper allowed for the participants to be blinded to the particular spice exposure, as well as specific intake amounts. Only one participant dropped out of the study, noting gastrointestinal side effects from first exposure to treatment, showing that this may be a feasible option for further study for many chronic pain sufferers. There may be potential dietary interactions of further relevance to study, such as a higher lipid/fat content in the meal with turmeric and black pepper for an increase in bioavailability. The utilization of the provision of the lunch meals to maintain diet intake consistency for the lunchtime meal over the study period helped to standardize these potential meal interactions across study weeks.

### 4.2. Limitations

However, the study also had some limitations. The majority of the study participants were middle-aged to older females as noted in Table 2. While previous reports indicate chronic pain is more prevalent in women than men, with higher prevalence at increasing age [2,45,46], the results from the current study, with the most prevalent demographic reporting chronic pain, cannot address whether sex differences may be present for dietary intervention responses across the life course. The study design did not include a placebo encapsulation group of an alternative spice or non-nutritive filler, which could be utilized with larger sample sizes or longer study durations in future studies. While the study did include a one-week ‘washout’ between the two exposure arms, the self-reported pain ratings varied in the magnitude of change from baseline across each week in the study. Those allocated to the treatment group of turmeric alone in week 1 changed (−1.55) from baseline, and those allocated to the treatment group of turmeric with black pepper in week 1 changed (−2.21) from baseline. During the ‘washout period’ in week 2, participants allocated to receive turmeric alone in week 1 reported a (−2.59) change from baseline, and participants allocated to receive turmeric with black pepper in week 1 reported a (−2.70) change from baseline. During the final week of the study (week 3), participants who received turmeric with black pepper had a difference of (−2.74) from baseline, and those who received turmeric alone in week 3 had a difference of (−3.05) from baseline. The ‘washout period’ (week 2) for all participants showed that, on average, NPRs were still reduced from baseline reported values, raising the potential of a sustained benefit of the initial week exposure for a duration beyond the acute effects that were observed. Future studies could be designed to test the duration of any pain-modifying effects once the dietary exposure or treatment has ceased, utilizing the ESM NPR approach. Similarly, effects of turmeric and black pepper tested within a design with a no-spice control could utilize single exposure versus chronic exposures with multi-day or multi-week meal consumption to further clarify any cumulative effects, which may be informative for assessing clinically meaningful changes within the chronic pain population from a dietary pattern perspective [39]. Previous research has shown high consistency in pain stability without intervention over a four-week period, indicating that small changes of 1 point from baseline may be clinically meaningful [38]. The modest sample size may have impacted our ability to discriminate statistically significant differences between the groups, supporting the need for further research addressing diverse types of chronic pain with larger sample sizes. The curcuminoid or terpenoid content of the commercially available spices was estimated based on published study comparisons but was not directly measured. Moreover, in contrast with herbal extracts that are utilized as supplements, the ground spice form of both turmeric and black pepper contains multiple other bioactive components beyond those of primary interest (curcuminoids and piperine). Processing of the spices, as well as meal preparation methods, such as exposure to heat and other chemical environments, could likewise influence bioactive compound concentrations. The encapsulated forms of the ground spices thus delivered a culinarily relevant amount, but they were non-cooked forms of both turmeric and black pepper. Whether or how these factors affect both the amount and bioavailability of specific phytochemicals was beyond the scope of the present study, while the effects on participants’ self-reported pain support future investigations in this context. Whether other dietary factors, such as lipids from breakfast, dinner, or snacks, as well as beverage consumption during meals, could have had an interaction with the turmeric and black pepper absorption would require a more invasive and rigorous design through a controlled feeding study. Finally, we are not able to discriminate whether variation in specific types and anatomic location of pain could have influenced individual pain responses with the current study size and design, although the anatomic location of chronic pain indicated by participants aligns with the most commonly reported back, hip, and knee [45]. As with other nutrition- and pain-relieving-focused interventions, individual subject response (including ‘responders’ and ‘non-responders’) variability could be investigated more fully with a large sample size to identify characteristics of populations most likely to benefit from this or other low-cost and low-burden interventions. Whether other spices may be beneficial (such as cinnamon and ginger, which have been tested for use in pain relief but were not tested here) warrants future exploration of these topics [10,12].

## 5. Conclusions

While this study demonstrates that culinary amounts of turmeric may be effective in reducing pain over time, efforts toward precision nutrition will benefit from tailored assessments as to what amounts (and under what conditions) may be best for each individual. As spices are most often consumed in combination with other spices within a single dish or meal, rather than in isolation, these results support the possibility of exploration of additional herb and spice consumption, particularly in the form of blends/mixtures, to provide a unique understanding of ways to increase diet quality in parallel with health benefits.

## Figures and Tables

**Table 1:** Inclusion and exclusion criteria for enrollment.

Inclusion Criteria	Exclusion Criteria
Gender: Any	Turmeric or black pepper allergy or any allergy that would prevent menu item selections for seven days of meals
Age: 40 or older	Recent change in pain levels, e.g., acute injury
Ethnicity: Any	Known eating disorder as previously diagnosed by a healthcare provider
BMI: 20–40 kg/m^2^	Pregnancy, anticipating pregnancy or lactation
Diet: No dietary prohibitions/allergies	No access to a phone capable of text messaging or an electronic device (phone, tablet, computer/laptop) with email and internet access
21-day availability	Inability to continue on protocol for three consecutive weeks
Moderate chronic pain rating (4–7 on 0–10 scale)	Medical conditions or medications that would prevent the ability to comply with treatment assignment (e.g., anti-coagulant medications, arrhythmia control medications, prescription narcotic pain medications)
	Cold, flu, virus, allergy or other condition that impairs sensory perceptions (taste and smell) that may impact protocol compliance; COVID19–positive status at time of enrollment
	Consumes an average of ≥1 serving of turmeric per day or has never consumed and/or would not be willing to consume turmeric; consumes black pepper ≥3 servings per day and/or would not be willing to abstain from pepper consumption for the prescribed days during the study; abstinence from or dislike of either turmeric or black pepper

Abbreviations: kg, kilogram; m, meter.

**Table 2:** Baseline data of participants.

Variable	Baseline Data
Sex (F/M) [% of sample]	27/2; [93.1%]/[6.9%]
Age (years)	54.34 ± 6.8 *
BMI (kg/m^2^)	35.31 ± 5.5 *
Overall baseline numeric pain ratings (scale: 0–10)	4.3 ± 2.2 *

Footnote: * mean ± standard deviation. Participants included in analysis, *n* = 29, due to one participant dropping from the study related to complaints of gastrointestinal distress. Abbreviations: F, female; M, male; BMI, body mass index; kg, kilogram; m, meter.

## Data Availability

The raw data supporting the conclusions of this article will be made available by the authors on request.

## Abbreviations

The following abbreviations are used in this manuscript:

BP	Black Pepper
SD	Standard Deviation
SE	Standard Error
